# Cardioprotective effects of early intervention with sacubitril/valsartan on pressure overloaded rat hearts

**DOI:** 10.1038/s41598-021-95988-3

**Published:** 2021-08-16

**Authors:** Xiaofei Li, Julie Braza, Ulrike Mende, Gaurav Choudhary, Peng Zhang

**Affiliations:** 1 Cardiovascular Research Center, Lifespan Cardiovascular Institute, Rhode Island Hospital, Providence, RI USA; 2 Vascular Research Laboratory, Providence VA Medical Center, Providence, RI USA; 3 Department of Medicine, Alpert Medical School of Brown University, Providence, RI USA

**Keywords:** Cardiovascular biology, Preclinical research, Translational research

## Abstract

Left ventricular remodeling due to pressure overload is associated with poor prognosis. Sacubitril/valsartan is the first-in-class Angiotensin Receptor Neprilysin Inhibitor and has been demonstrated to have superior beneficial effects in the settings of heart failure. The aim of this study was to determine whether sacubitril/valsartan has cardioprotective effect in the early intervention of pressure overloaded hearts and whether it is superior to valsartan alone. We induced persistent left ventricular pressure overload in rats by ascending aortic constriction surgery and orally administrated sacubitril/valsartan, valsartan, or vehicle one week post operation for 10 weeks. We also determined the effects of sacubitril/valsartan over valsartan on adult ventricular myocytes and fibroblasts that were isolated from healthy rats and treated in culture. We found that early intervention with sacubitril/valsartan is superior to valsartan in reducing pressure overload-induced ventricular fibrosis and in reducing angiotensin II-induced adult ventricular fibroblast activation. While neither sacubitril/valsartan nor valsartan changes cardiac hypertrophy development, early intervention with sacubitril/valsartan protects ventricular myocytes from mitochondrial dysfunction and is superior to valsartan in reducing mitochondrial oxidative stress in response to persistent left ventricular pressure overload. In conclusion, our findings demonstrate that sacubitril/valsartan has a superior cardioprotective effect over valsartan in the early intervention of pressure overloaded hearts, which is independent of the reduction of left ventricular afterload. Our study provides evidence in support of potential benefits of the use of sacubitril/valsartan in patients with resistant hypertension or in patients with severe aortic stenosis.

## Introduction

Left ventricular (LV) remodeling due to pressure overload is common in many cardiovascular diseases such as systemic hypertension and aortic stenosis and is associated with increased morbidity and mortality^1^. Cardiac myocytes and fibroblasts are two major cell types in the myocardium and play a key role in determining the remodeling response and cardiac function. Upon chronic LV pressure overload, cardiac myocytes become hypertrophied and change their cellular properties, whereas cardiac fibroblasts convert into “activated” myofibroblasts, proliferate and/or increase deposition of extra cellular matrix. Ultimately, these pathological changes in myocardium result in the development of heart failure and poor prognosis^2,3^.

Sustained activation of endogenous neurohormonal systems, particularly the renin–angiotensin–aldosterone system (RAAS), is a key feature in the occurrence and development of pressure overload-induced cardiomyopathy^4^ and a target of current pharmacologic interventions (e.g., angiotensin-converting enzyme [ACE] inhibitors, angiotensin AT_1_ receptor blockers, and aldosterone antagonists). While these traditional interventions have beneficial effects^4,5^, new therapeutic strategies are needed to protect the heart from pressure overload-induced cardiac injury. This is especially important in progressive fixed afterload states such as from aortic stenosis when surgical and transcatheter interventions are not yet indicated (e.g., lack of early signs of ventricular dysfunction or onset of symptoms)^6^ or from resistant hypertension. Mitigating adverse remodeling and maintaining optimal cardiac function in such settings have the potential to improve outcomes in these populations.

Sacubitril/valsartan is the first-in-class Angiotensin Receptor Neprilysin Inhibitor (ARNi), whose mode of action involves AT_1_ receptor blockade and neprilysin inhibition^7^. With oral administration, sacubitril/valsartan dissociates into valsartan and sacubitril (neprilysin inhibitor prodrug), which is then metabolized to the active neprilysin inhibitor sacubitrilat (also known as LBQ657)^7^. Natriuretic peptides counterbalance RAAS and promote vasodilation, natriuresis, and diuresis^8^. They also have anti-inflammatory, antifibrotic, and antihypertrophic effects in myocardium and cardiac cells^9–11^. During cardiac disease progression, responsiveness to natriuretic peptides decreases primarily due to increased natriuretic peptide degradation by neprilysin^12^. Neprilysin inhibition thus has emerged as a pharmacological approach to reduce degradation of endogenous natriuretic peptides and thereby increases their protective effects. However, neprilysin inhibition alone did not show promising efficacy in clinical trials^13^, possibly because it also reduced degradation of angiotensin II (Ang II) and other vasoconstrictor peptides^14^, which gave rise to the drug combination that can simultaneously inhibit neprilysin and block RAAS and produce combined beneficial effects. Importantly, the PARADIGM-HF trial revealed that sacubitril/valsartan reduces mortality, rehospitalization, and symptoms in heart failure patients more than enalapril^15^, suggesting that the combination of AT_1_ receptor blocker and neprilysin inhibitor has a greater therapeutic effect than RAAS inhibition alone.

To date, however, clinical and animal studies have mainly focused on the beneficial effects of sacubitril/valsartan in the settings of heart failure^15–19^. While there were a few studies about sacubitril/valsartan in hypertension^20,21^, the major objective of these studies was to determine the efficiency of sacubitril/valsartan in lowing systemic blood pressure. In this study, we hypothesized that sacubitril/valsartan has cardioprotective effect in the early intervention of pressure overloaded hearts and is superior to valsartan. We used a LV pressure overload rat model that was induced by ascending aortic constriction (AAC) to mimic aortic stenosis or resistant hypertension and orally administrated sacubitril/valsartan, valsartan, or vehicle to rats one week post operation for 10 weeks. We determined cardiac remodeling including cardiac hypertrophy and fibrosis and assessed mitochondrial function and oxidative stress in cardiac myocytes. Together with our in vitro studies, our findings revealed a superior cardioprotective effect of sacubitril/valsartan over valsartan alone against oxidative stress, mitochondrial dysfunction, and cardiac fibrosis in the hearts with persistent pressure overload, which indicates that early intervention with sacubitril/valsartan might be clinically useful in protecting hearts and potentially improving prognosis in patients with aortic stenosis or in patients with resistant hypertension.

## Materials and methods

All animal studies were carried out in compliance with the NIH Guide for the Care and Use of Laboratory Animals and the ARRIVE guidelines and were approved by the Institutional Animal Care and Use Committee of Rhode Island Hospital. Animals were housed in a pathogen-free animal facility with an automated 12-h light/dark cycle and have access to regular chow and water ad libitum. All efforts were made to minimize suffering.

### Ascending aortic constriction (AAC) and drug treatments

Four to five weeks old male Sprague–Dawley rats (Charles River Laboratories, Wilmington, MA) were subjected to LV pressure overload by AAC via placing a vascular clip on the ascending aorta^22–24^. Briefly, animals were sedated with 3–5% isoflurane, endotracheally intubated using an 18-gauge catheter, and mechanically ventilated at 95 breaths per minutes with a tidal volume of 0.5 ml of 100% oxygen supplemented with 1.5–2% isoflurane. Lidocaine (2 mg/kg) was injected subcutaneously as a local anesthetic. A 1-cm incision was made to the right chest wall at the first intercostal space and the thoracic cage was approached. The thymus was pushed aside, and the ascending aorta was exposed and dissected, followed by placing a small titanium clip (Teleflex, Morrisville, NC) around the ascending aorta with consistent constriction. The chest cavity was closed in layers using 4–0 Vicryl suture and the animals were recovered. The same procedure without clip placement was performed in sham-operated controls (Sham). Buprenorphine (0.03 mg/kg) was administrated subcutaneously for analgesia twice a day for 3 days. One-week post-surgery, the AAC animals were randomized into 3 groups for 10-week treatment with vehicle (water), valsartan (31 mg/kg, Novartis Pharmaceuticals [Novartis]), or sacubitril/valsartan (68 mg/kg, Novartis) via oral gavage once per day. Sacubitril/valsartan was administrated at 68 mg/kg/day by oral gavage to achieve adequate pharmacologic effect^7^. Valsartan was administrated at 31 mg/kg/day to correspond to the dose of valsartan delivered by sacubitril/valsartan^7^. AAC- and sham-operated animals administrated with vehicle (water) served as controls. After animals were sacrificed at the end of the experiments, the aorta clips were collected for measurement of their internal areas to confirm the comparable degree of the aorta constriction among AAC animals.

### Echocardiographic measurements

Transthoracic echocardiography was performed after 4-week and 10-week treatment using a Vevo2100 (Fujifilm VisualSonics, Toronto, Canada) echocardiography system with a MS250 probe as we described previously^25^. Animals were lightly anesthetized using 1.5–2% isoflurane. Body temperature was kept at 37 °C. Electrocardiogram, respiration, and rectal temperature were continuously monitored with an integrated physiology platform (Fujifilm VisualSonics). 2D images in the LV parasternal long axis views and M-mode images in the short-axis views at the mid-papillary level were obtained to assess LV dimensions and wall thickness. LV inflow velocities (E and A wave velocities) were interrogated by conventional pulse-wave Doppler from the apical four chamber view with the sample volume placed at the tip of the mitral valve leaflets. The mitral annulus longitudinal velocities (E’ and A’) were determined by pulse-wave tissue Doppler from the apical four-chamber view with the sample volume placed at the septal side of the mitral annulus. The staff was blinded from the treatment groups. Measurements were averaged from three consecutive beats during expiration.

### Tissue harvest and ventricular fibrosis analysis

Animals were euthanized for tissue harvest after 10-week treatment. Body weights, heart weights, ventricular weights, and tibia lengths were obtained. The ventricles were formalin fixed, paraffin-embedded, and cross-sectioned at 6 µm thickness. To assess fibrosis, ventricular cross-sections were deparaffinized, rehydrated, and stained with Picrosirius Red^25,26^. Entire cross-sections were then scanned using an Aperio Scanscope® CS System (Leica Biosystems, Buffalo Grove, IL) and analyzed using ImageJ software as we described previously^25^. The percentage of fibrosis (Sirius Red positive area/total tissue area × 100) was then calculated. For all hearts, same regions were used for imaging and analysis.

### Mitochondrial respiration measurement in ventricular myocytes from pressure overloaded hearts

To assess mitochondrial respiration in ventricular myocytes from pressure-overload hearts, adult rat ventricular myocytes were isolated by retrograde heart perfusion as we described previously^25,27^ and were plated in laminin (20 µg/ml, Life Technologies, Carlsbad, CA)-coated Seahorse XFe96 V3 PS cell culture microplates (Agilent, Santa Clara, CA). Mitochondrial respirations were measured in the next day using Agilent Seahorse XFe96 Analyzer according to the manufacturer's protocol (Agilent). Briefly, hydrated sensor cartridge was loaded in its injection ports with oligomycin (Sigma, St. Louis, MO), carbonyl cyanide-p-trifluoromethoxyphenylhydrazone (FCCP, Sigma), and a mix of rotenone and antimycin A (Sigma), respectively. Seahorse XF DMEM Medium (pH 7.4, Agilent) supplemented with 10 mM Glucose, 1 mM Pyruvate, and 2 mM L-Glutamine (Sigma) was used as assay medium. Mitochondrial respiration was measured in Agilent Seahorse XFe96 Analyzer using the following protocol: 3 × [2 min mix, 2 min wait, 2 min measure] for baseline and after injection of oligomycin, FCCP, and a mix of rotenone and antimycin A, respectively. After the assays, cells were stained with Hoechst 33342 (1 µg/ml, Thermo-Fisher) for 15 min at 37 °C and fluorescent signals were measured with excitation/emission at 350/460 nm using a plate reader SynergyMx with Gen5 software (BioTek, Winooski, VT) for cell number normalization.

### Mitochondrial superoxide measurement in ventricular myocytes from pressure overloaded hearts

Adult rat ventricular myocytes from pressure overloaded hearts were plated in laminin (10 µg/ml)-coated glass-bottom dishes (Matsunami, Bellingham, WA) with Medium 199 (Sigma) supplemented with 2 mg/ml BSA, 2 mM L-carnitine, 5 mM creatine, 5 mM taurine, 0.1 µM insulin, 100 U/ml penicillin, and 100 mg/ml streptomycin^28^. In the next day, the fluorescent probe MitoSOX™ Red (Life Technologies, 5 µM) was used to measure mitochondrial superoxide according to the manufacturer's protocol. Images (20X and 60X) of myocytes were taken on a Nikon Ti-E Spinning Disk confocal microscope (Nikon Instruments, Melville, NY) with an sCMOS camera (Andor Zyla 4.2) using an argon laser excitation at 488 nm and emission collection through a 625/90 nm filter. For the representative images in figures, intensity of images was uniformly and linearly adjusted to maintain relative differences in fluorescence. At least 10 images (20X) for each condition (140–200 myocytes from each in vitro treatment and 100–200 myocytes from each in vivo treated animal) were acquired and quantitated. The mean values of the whole cell fluorescence of MitoSOX™ Red were obtained using NIS-Elements software 3.2.

### Treatment of adult rat ventricular myocytes and fibroblasts from normal rats in culture

Adult rat ventricular myocytes and fibroblasts were isolated from six to eight weeks old male Sprague–Dawley rats (Charles River Laboratories) without any surgery as we previously described^27^. Rod-shaped adult ventricular myocytes were plated into laminin (10 µg/ml)-coated culture dishes and cultured with Medium 199 supplemented with 2 mg/ml BSA, 2 mM L-carnitine, 5 mM creatine, 5 mM taurine, 0.1 µM insulin, 100 U/ml penicillin, and 100 mg/ml streptomycin^28^. Because the compound of sacubitril/valsartan consists of the inactive prodrug sacubitril that has no effect on cells in culture^29^, a combination of LBQ657 (the active form of sacubitril) and valsartan was used in culture to mimic sacubitril/valsartan and compared to valsartan alone. After overnight recovery, myocytes were preincubated with LBQ657 (10 µM, Novartis), valsartan (0.3 or 1 µM, Novartis), or the combination of valsartan (0.3 or 1 µM) and LBQ657 (10 µM, Novartis) for 1 h before stimulation with angiotensin II (Ang II, 1 µM, Sigma). For ^14^C-phenylalanine protein incorporation and mRNA expression (see below), myocytes were treated for 72 h and all drugs were replenished every 24 h by medium changes. For determinations of mitochondrial superoxide, myocytes were treated for 24 h, followed by MitoSOX™ Red labelling and confocal imaging as described above.

Adult ventricular fibroblasts were cultured in DMEM/F12 (Life Technologies) with 10% fetal bovine serum, 100 U/ml penicillin, and 100 mg/ml streptomycin. The high purity of our fibroblast preparations was characterized and demonstrated previously^27^. All experiments were performed on Passage 1 fibroblasts. After being serum-starved for 24 h, fibroblasts were preincubated with LBQ657 (10 µM), valsartan (0.3 or 1 µM), or the combination of valsartan (0.3 or 1 µM) and LBQ657 (10 µM) for 1 h before stimulation with Ang II (1 µM) for 72 h.

### ^14^C-phenylalanine protein incorporation in adult ventricular myocytes

To assess protein synthesis in adult rat ventricular myocytes, cellular proteins were continuously labelled with ^14^C-phenylalanine (0.05 µCi/ml, > 450 mCi/mmol; ICN) during the drug treatments in culture. On the assay day, myocytes were washed with 1 × PBS, followed by precipitation of cellular protein with 10% trichloroacetic acid for at least 1 h at 4 °C. Cellular proteins were washed once with 10% trichloroacetic acid, solubilized in 1% SDS for 1 h at 37 °C, and then quantified by liquid scintillation counting (Beckman Coulter LS6500). The radioactive counts were normalized to cell numbers.

### Real-time PCR for mRNA expression

Total RNA from adult myocytes or fibroblasts was extracted using Trizol (Life Technologies). cDNA was produced using TaqMan Reverse Transcription reagents (Life Technologies), and real-time PCR was performed using universal PCR master mix with FAM-labeled TaqMan probes (Life Technologies) for atrial natriuretic factor (ANF), alpha smooth muscle actin (α-SMA), and 18S. Each sample was assayed in duplicate in two independent PCR reactions and normalized to 18S expression. Samples without a template during PCR served as negative controls. PCR cycling was performed at 95 °C for 10 min, followed by 95 °C for 15 s and 60 °C for 1 min for a total of 40 cycles using Applied Biosystems® ViiA™ 7 Real-Time PCR System (Life Technologies). Assay results were analyzed using sequence detecting system software (SDS version 2.3, Life Technologies).

### MTS cell proliferation assays in adult ventricular fibroblasts

Fibroblast proliferation was assessed using CellTiter 96® AQ_ueous_ One Solution Cell Proliferation Assay kit (Promega, Madison, WI). Passage 1 fibroblasts were seeded into 96-well plate at a density of 20,000 cells/well. After treatment with drugs for 72 h (see fibroblast culture above), 20 µl/well of CellTiter 96® AQ_ueous_ One Solution Reagent was added to each well. The plates were incubated for 4 h at 37 °C, and absorbance at 490 nm was measured using a plate reader SynergyMx with Gen5 software (BioTek).

### Statistical analysis

N refers to number of rats in the in vivo study and n refers to in vitro determinations. Statistical differences were assessed by unpaired, two-tailed Student’s t test or two-way ANOVA followed by Bonferroni post-tests for comparison of individual means. A *P* value of < 0.05 was considered statistically significant.

## Results

### Validation of the AAC rat model and LV pressure overload among all AAC experimental groups

**Figure 1:**
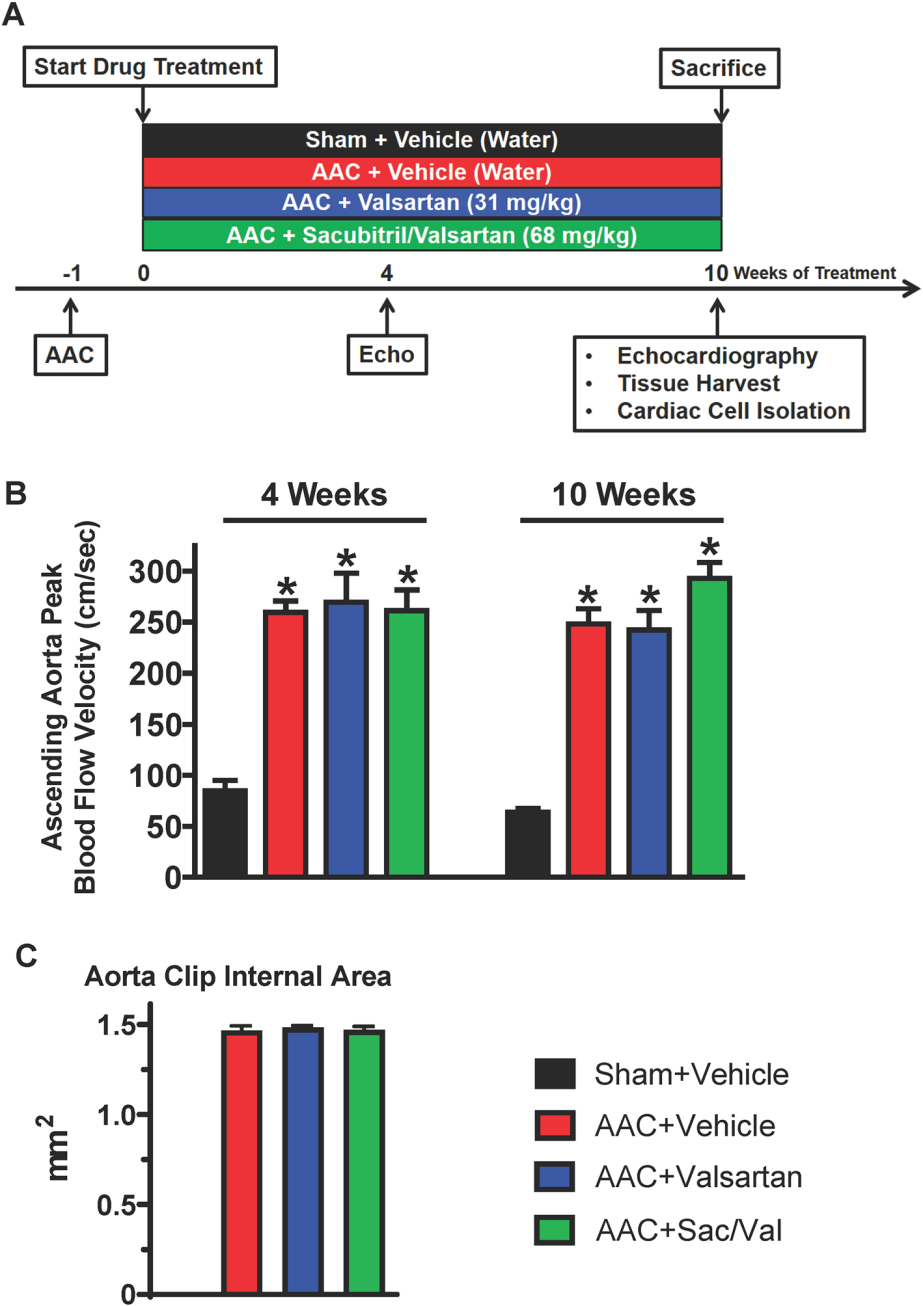
Validation of the AAC rat model and LV pressure overload among AAC experimental groups. (**A**) Experimental design for in vivo treatments: rats were subjected to AAC or sham operation 1 week before treatment with vehicle, valsartan, or sacubitril/valsartan at indicated doses by oral gavage for 10 weeks. Echocardiographic measurements were performed at indicated time points, followed by tissues harvest or cardiac cell isolation after 10-week treatment. (**B**) Ascending aorta peak blood flow velocity after 4-week (*left*) and 10-week (*right*) treatment. Mean ± SEM. N = 8–11 rats per group. *P < 0.05 versus respective Sham + Vehicle. (**C**) The internal area of the aorta clips from different AAC groups. Mean ± SEM. N = 8–11 rats per group.

We performed AAC surgery to induce LV pressure overload in adult rats, for which a vascular clip was placed on the ascending aorta. Since the size of the clip on the ascending aorta is fixed and the aorta cannot expand as the rats grow in size, LV pressure overload progressively increases and persists over the course of the study. One week post-surgery, AAC animals were randomized into 3 groups for 10-week treatment with vehicle (water), valsartan (31 mg/kg), or sacubitril/valsartan (68 mg/kg). Sham-operated animals without clip placement and administrated with water were included as a control group. As shown in Fig. 1A, four experimental groups included sham-operated rats treated with water (sham + vehicle), AAC rats treated with water (AAC + vehicle), AAC rats treated with valsartan (AAC + valsartan), and AAC rats treated with sacubitril/valsartan (AAC + Sac/Val). We first assessed the ascending aorta peak blood flow velocity in all 4 experimental groups using the non-invasive echocardiography. As expected, constriction of the ascending aorta via AAC surgery resulted in a significant and reproducible increase in ascending aorta peak blood flow velocity in comparison to the sham-operation (Fig. 1B). Importantly, among the 3 AAC groups. there were comparable increases of ascending aorta peak blood flow velocity both after 4-week and 10-week treatment time points (Fig. 1B), indicating a similar and persistent increase of LV afterload among the AAC groups over the course of the study. In addition, at the end of the experiments, we collected the aorta clips from the AAC rats and measured their internal area. As shown in Fig. 1C, there was no difference in the internal areas of the aorta clips among AAC + vehicle group (1.47 ± 0.02 mm^2^), AAC + valsartan group (1.48 ± 0.01 mm^2^, *P* = 0.55 vs. AAC + vehicle), and AAC + Sac/Val group (1.47 ± 0.02 mm^2^, *P* = 0.92 vs. AAC + vehicle), further confirming the same degree of aortic constriction among AAC animals across treatment groups. Together, these characteristics validated not only the success in induction of LV pressure overload via AAC surgery in our hands but also the high consistency in LV pressure overload among all 3 AAC groups.

### Sacubitril/valsartan is superior to valsartan in reducing ventricular fibrosis, while both have no effects on ventricular hypertrophy development in response to persistent LV pressure overload

**Figure 2:**
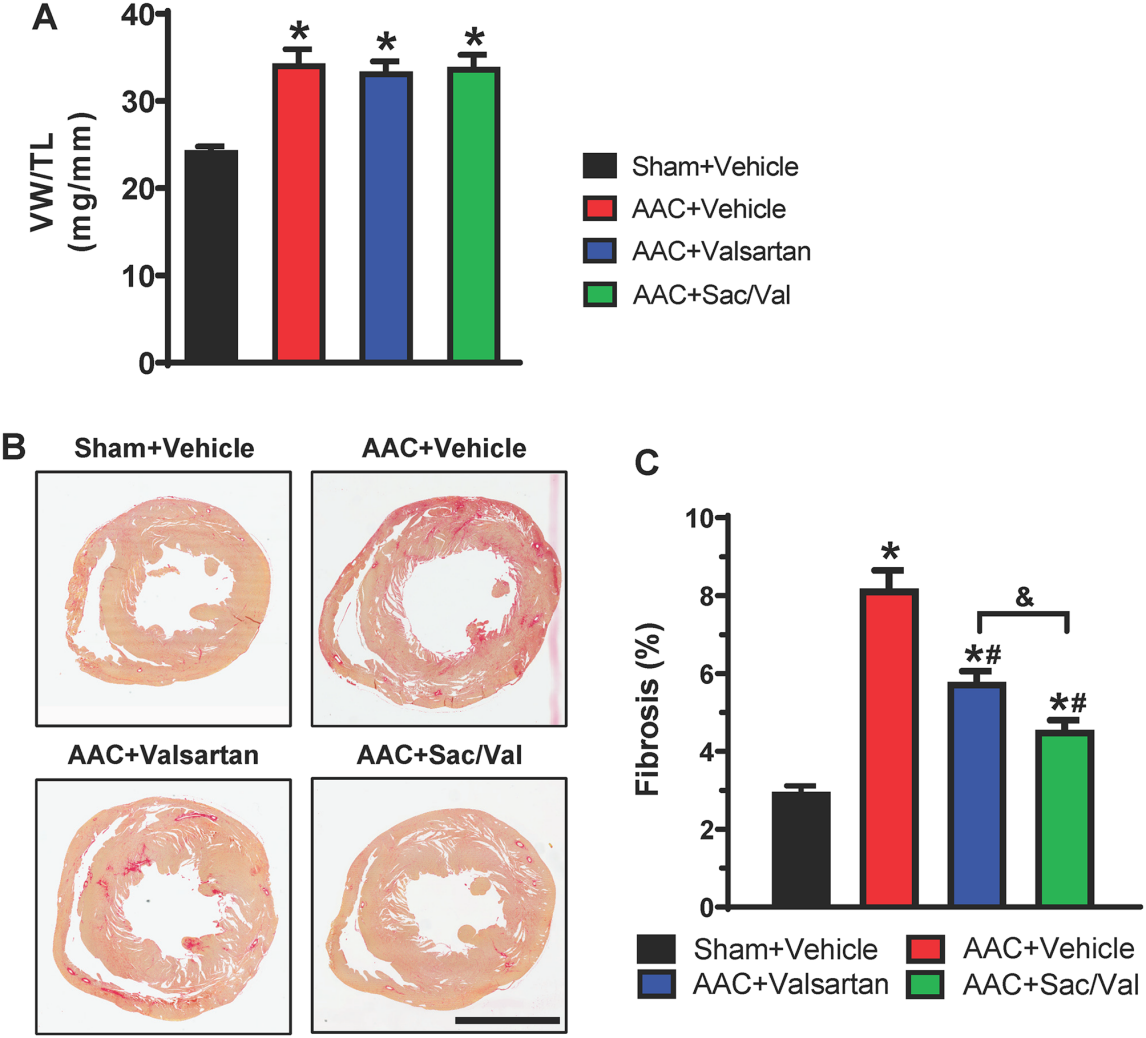
Sacubitril/valsartan is superior to valsartan in reducing ventricular fibrosis in response to persistent LV pressure overload. (**A**) Ventricular weight to tibia length ratios (VW/TL) after 10-week treatment. N = 8–11 rats per group. Mean ± SEM. *P < 0.05 versus Sham + Vehicle. (**B,C**) Representative images (**B**) and quantitation (**C**) of Picrosirius Red staining on ventricular cross-sections from indicated rats described in Fig. 1A. Scale bar: 5 mm. Mean ± SEM. N = 8–11 rats per group. *P < 0.05 versus Sham + Vehicle; ^#^*P* < 0.05 versus AAC + Vehicle; ^&^*P* < 0.05 for the indicated comparison.

Cardiac hypertrophy and fibrosis are two major features in cardiac remodeling response to chronic pressure overload. We thereby assessed the effects of sacubitril/valsartan and valsartan alone on the development of ventricular hypertrophy and fibrosis in response to AAC-induced persistent increase of LV pressure. Determined by ventricular weight to tibia length ratios (Fig. 2A and Supplementary Table S1 online), vehicle-treated AAC rats showed a 41% increase in ventricular weight than sham-operated rats at the end of the experiments, while there were no differences in ventricular weight to tibia length ratios in either sacubitril/valsartan- or valsartan-treated AAC groups compared to vehicle-treated AAC controls. Similar observations were obtained from echocardiographic measurements at both 4- and 10-week treatment time points (Table 1): in comparison to the sham-operated rats, AAC led to increases in LV wall thickness and LV ejection fraction (EF) and reductions in LV end systolic and diastolic volumes, and these phenotypic changes were comparable in all AAC groups regardless of treatments. These data suggested the development of concentric cardiac hypertrophy in response to persistent LV pressure overload over the course of the study, which was not altered by sacubitril/valsartan or valsartan treatment. We then assessed ventricular fibrosis development in AAC rats and the effects of sacubitril/valsartan or valsartan alone. As shown in Figs. 2B/C, a 2.8-fold increase of collagen deposition in ventricular tissue sections in the AAC + vehicle group compared to sham + vehicle controls was attenuated to 2.0-fold by valsartan and to a greater extent (1.5-fold) by sacubitril/valsartan treatment, suggesting that sacubitril/valsartan is superior to valsartan alone in mitigating cardiac fibrosis in pressure-overloaded hearts.

**Table 1:** Echocardiographic measurements of operated rats after 4- and 10-week treatments with vehicle (H_2_O), valsartan (31 mg/kg/day), and sacubitril/valsartan (Sac/Val, 68 mg/kg/day).

Parameter	Sham + Vehicle (N = 8)	AAC + Vehicle (N = 10)	AAC + Valsartan (N = 11)	AAC + Sac/Val (N = 11)
**After 4-week treatment**				
HR (bpm)	361 ± 10	347 ± 8	345 ± 10	351 ± 14
LVESV (µl)	66.2 ± 12.1	26.7 ± 7.6*	19.3 ± 4.8*	28.8 ± 23.9*
LVEDV (µl)	304.4 ± 19.8	244.2 ± 17.3*	219.6 ± 18.4*	217.2 ± 26.0*
EF (%)	73.7 ± 3.4	87.1 ± 1.9*	89.2 ± 1.8*	83.7 ± 2.7*
E/A	1.3 ± 0.1	1.4 ± 0.1	1.3 ± 0.1	1.2 ± 0.1
E/E′	− 21.1 ± 1.3	− 28.6 ± 2.0*	− 29.5 ± 2.4*	− 30.6 ± 3.8
IVSd (mm)	1.6 ± 0.2	2.4 ± 0.1*	2.2 ± 0.1*	2.3 ± 0.1*
LVPWd (mm)	2.3 ± 0.2	2.7 ± 0.2	2.6 ± 0.1	2.6 ± 0.2
LV Mass (mg)	819 ± 86	1092 ± 94	1017 ± 60	943 ± 102
**After 10-week treatment**				
HR (bpm)	333 ± 9	330 ± 9	342 ± 9	327 ± 6
LVESV (µl)	66.1 ± 10.3	52.0 ± 15.4	57.0 ± 23.9	43.6 ± 13.7
LVEDV (µl)	306.6 ± 19.4	300.4 ± 23.6	261.5 ± 31.1	276.2 ± 20.5
EF (%)	74.2 ± 2.6	79.4 ± 3.4*	82.8 ± 3.5*	84.0 ± 2.2*
E/A	1.4 ± 0.1	1.2 ± 0.1	1.3 ± 0.1	1.3 ± 0.1
E/E′	− 20.5 ± 1.7	− 26.8 ± 1.5*	− 28.2 ± 2.6*	− 27.3 ± 2.6
IVSd (mm)	1.8 ± 0.1	2.5 ± 0.1*	2.4 ± 0.1*	2.5 ± 0.2*
LVPWd (mm)	2.1 ± 0.2	2.5 ± 0.1*	2.5 ± 0.2	2.5 ± 0.2
LV Mass (mg)	1058 ± 28	1551 ± 141*	1372 ± 71*	1442 ± 69*

HR: heart rates; LV: left ventricle; LVESV: LV end-systolic volume; LVEDV: LV end-diastolic volume; EF: ejection fraction; E/A: ratio of the early to late mitral inflow velocities; E/E′: ratio between early mitral inflow velocity and mitral annular early diastolic velocity; IVSd: Interventricular septal at the end of diastole; LVPWd: Left ventricular posterior wall at the end of diastole. Mean ± SEM. *P < 0.05 versus Sham + Vehicle.

### Sacubitril/valsartan has a modest effect on Ang II-induced adult ventricular myocyte hypertrophy and is superior to valsartan in reducing Ang II-induced adult ventricular fibroblast activation in culture

**Figure 3:**
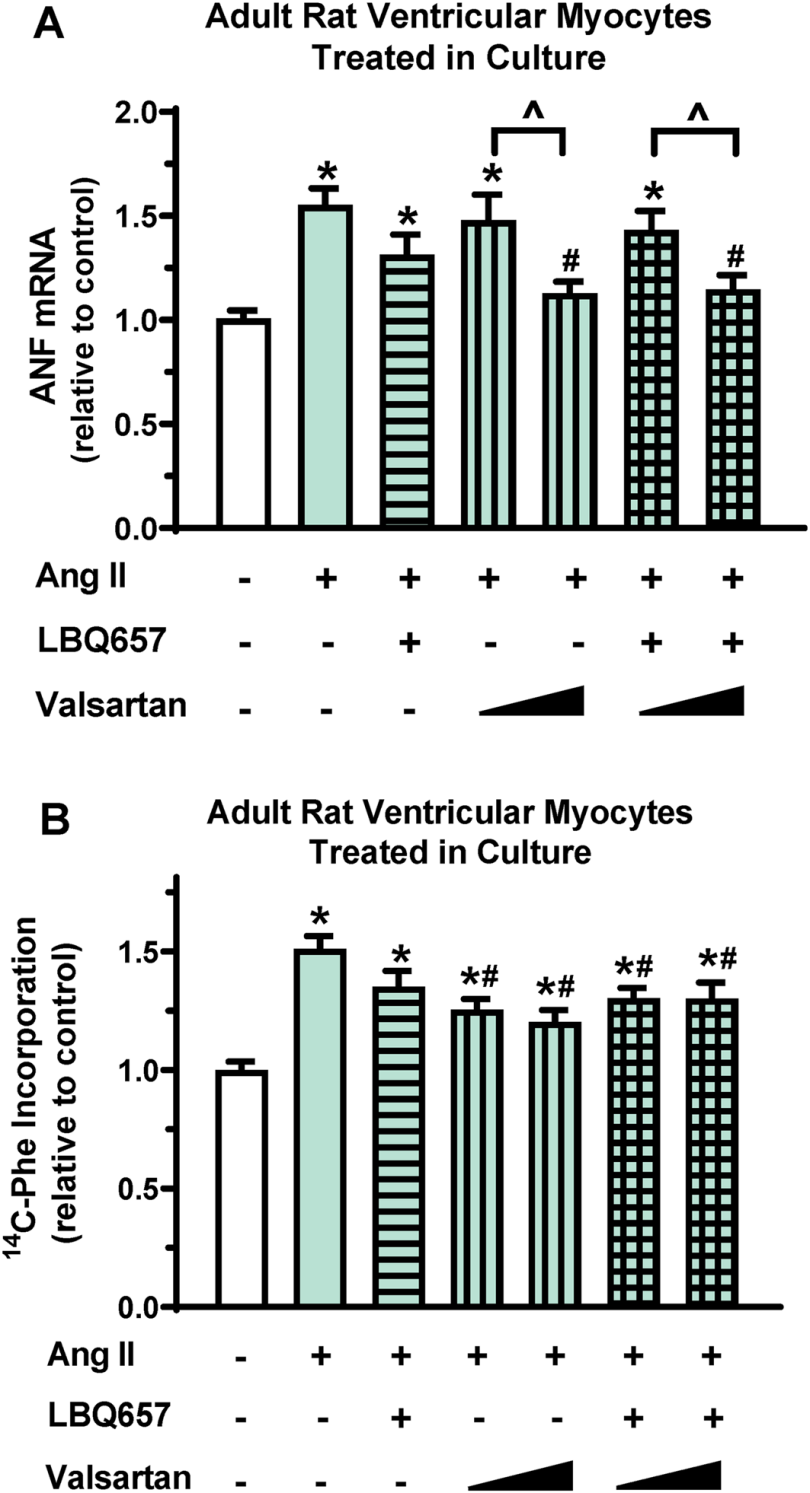
Sacubitril/valsartan has a modest effect on Ang II-induced adult ventricular myocyte hypertrophy in culture. Adult rat ventricular myocytes were pretreated with LBQ657 (10 µM), valsartan (0.3 or 1 µM) alone or the combination of LBQ657 (10 µM)/valsartan (0.3 or 1 µM) for 1 h before Ang II stimulation (1 µM, 72 h). Ang II and drugs were replaced every 24 h in myocyte treatments. (**A**) ANF mRNA expression normalized to 18S and expressed relative to vehicle controls (open bar, without Ang II, valsartan, and LBQ657). n = 9 determinations per group. (**B**) ^14^C-Phenylalanine incorporation normalized to cell numbers and expressed relative to vehicle controls. n = 10–12 determinations per group. Mean ± SEM. *P < 0.05 versus Control; ^#^*P* < 0.05 versus Ang II; ^*P* < 0.05 Valsartan at 1 µM versus 0.3 µM.

Using primary adult ventricular myocytes from healthy, untreated rats, we further performed experiments to determine whether sacubitril/valsartan could directly regulate myocyte hypertrophy in culture. As showed in Fig. 3, Ang II markedly increased ANF mRNA expression and protein synthesis indicated by ^14^C-phenylalanine incorporation in ventricular myocytes, suggesting an induction of ventricular myocyte hypertrophy by Ang II stimulation in culture. Valsartan dose-dependently inhibited Ang II-induced ANF mRNA expression and 1 µM valsartan almost completely attenuated Ang II-induced increase in ANF mRNA expression (Fig. 3A). In contrast, valsartan at both 0.3 and 1 µM significantly reduced ^14^C-phenylalanine incorporation, but the inhibitory effects appeared to be partial with no significant difference between the two doses tested (Fig. 3B). The combination of valsartan and LBQ657 did not further enhance the inhibitory effects of valsartan alone on either Ang II-induced ANF mRNA expression or protein synthesis (Fig. 3). These findings suggest that sacubitril/valsartan and valsartan alone have comparable and modest effects on adult ventricular myocyte hypertrophy in culture.

**Figure 4:**
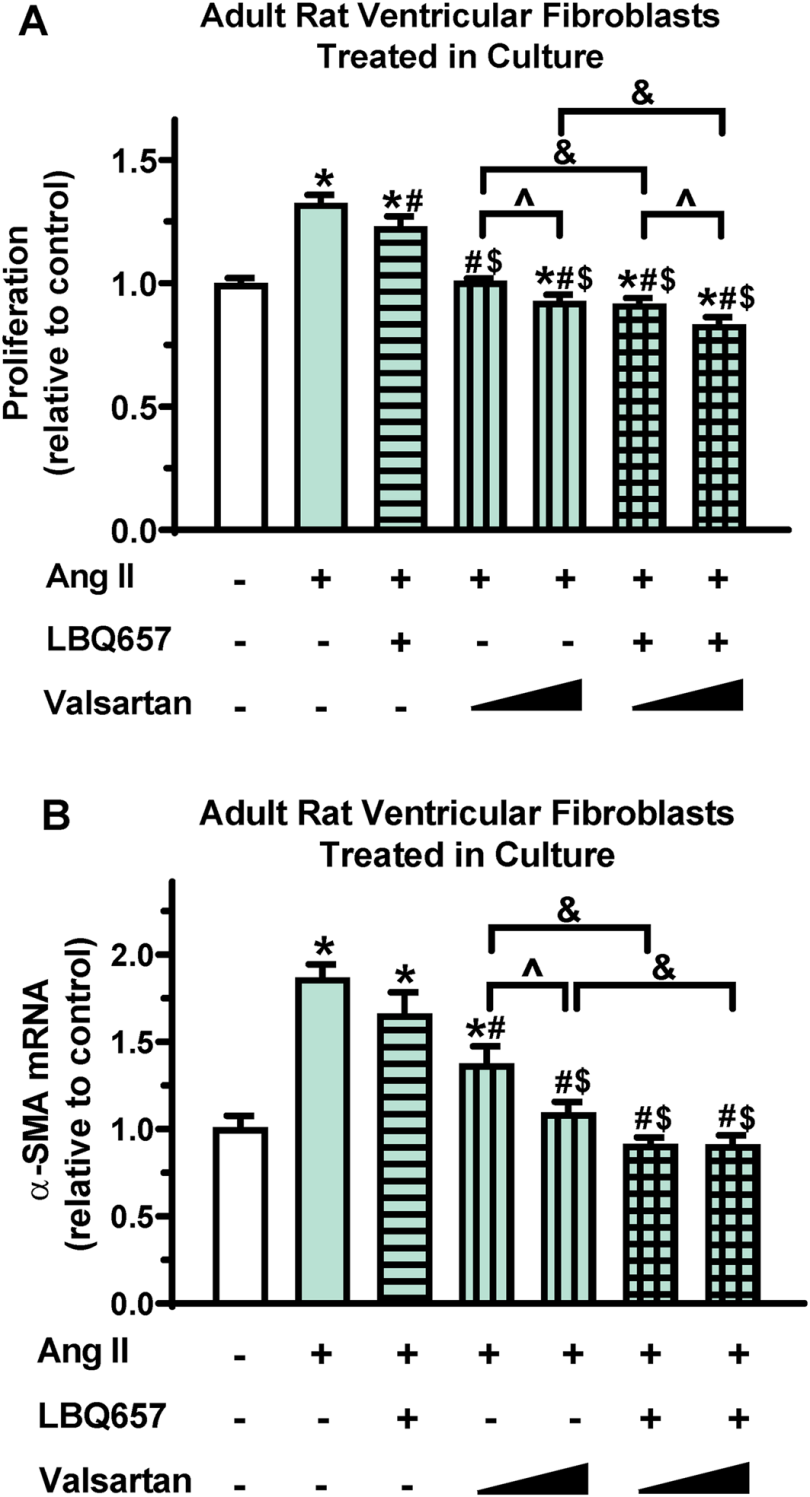
Sacubitril/valsartan is superior to valsartan in reducing Ang II-induced adult ventricular fibroblast activation in culture. Adult rat ventricular fibroblasts were pretreated with LBQ657 (10 µM), valsartan (0.3 or 1 µM) alone or the combination of LBQ657 (10 µM) and valsartan (0.3 or 1 µM) for 1 h before Ang II stimulation (1 µM, 72 h). (**A**) MTS cell proliferation assays normalized to vehicle controls (open bar, without Ang II, valsartan, and LBQ657). n = 8 determinations per group. (**B**) α-SMA mRNA expression normalized to 18S and expressed relative to vehicle controls. n = 6 determinations per group. Mean ± SEM. *P < 0.05 versus Control; ^#^*P* < 0.05 versus Ang II; ^$^*P* < 0.05 versus Ang II + LBQ657; ^*P* < 0.05 Valsartan at 1 µM versus 0.3 µM; ^&^*P* < 0.05 for the indicated comparison.

During cardiac fibrosis development, cardiac fibroblast activation plays a key role by proliferating quickly and expressing excess stress fibers (e.g., α-SMA)^30,31^. We then determined whether the effects of sacubitril/valsartan on cardiac fibrosis may result from its direct regulation on cardiac fibroblast activation using adult ventricular fibroblasts isolated from healthy rats and stimulated in culture with Ang II, a well-known pro-fibrotic agonist for fibroblast activation. As expected, Ang II stimulation induced activation of adult ventricular fibroblasts, evidenced by increased fibroblast proliferation (Fig. 4A) and α-SMA expression (Fig. 4B). Valsartan dose-dependently inhibited Ang II-induced fibroblast activation, while the inhibitory effects of LBQ657 alone were moderate (Fig. 4). Importantly, the combination of valsartan and LBQ657 had greater inhibitory effects on both fibroblast proliferation and α-SMA expression than valsartan alone (Fig. 4), suggesting that sacubitril/valsartan is superior to valsartan alone in inhibiting cardiac fibroblast activation.

### Sacubitril/valsartan protects ventricular myocytes from mitochondrial dysfunction in response to persistent LV pressure-overload

**Figure 5:**
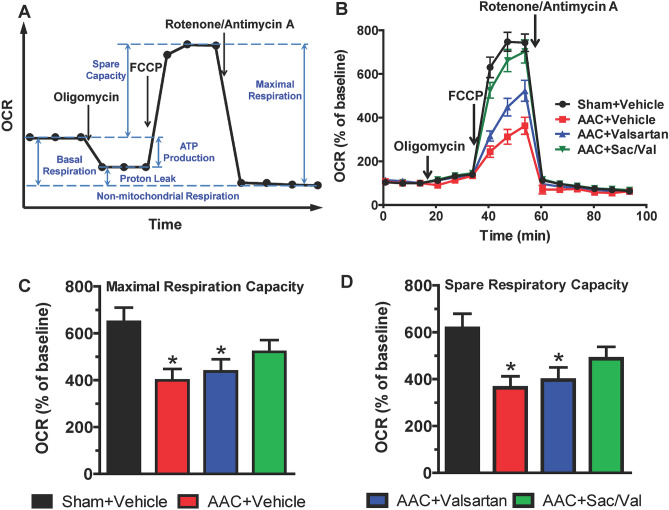
Sacubitril/valsartan protects ventricular myocytes from mitochondrial dysfunction in response to persistent LV pressure-overload. (**A**) Typical scheme of Seahorse XFe Cell Mito Stress Test for oxygen consumption rate (OCR) measurements in cells. (**B**) Representative OCR measured in adult rat ventricular myocytes of indicated groups after 10-week treatment. OCR were normalized to cell numbers and expressed relative to baseline. Sac/Val: sacubitril/valsartan. Mean ± SEM. n = 15–19 determinations from one rat per group. (**C**) Maximal respiration capacity and (**D**) Spare respiratory capacity that were normalized to cell numbers and expressed relative to baseline. For each rat, n = 15–19 determinations were averaged as one biological sample. Mean ± SEM. N = 6–7 rats per group. * *P* < 0.05 versus Sham + Vehicle.

Mitochondria through oxidative phosphorylation are the major source of energy in the hearts^32^. Patients with heart failure showed a reduction of mitochondrial function in cardiac tissue, indicated by the low capacity in mitochondrial oxygen consumption^33–35^. We thus asked whether mitochondrial dysfunction in oxidative phosphorylation occurred already in ventricular myocytes in response to persistent LV pressure overload and whether sacubitril/valsartan is protective. To that end, we performed real-time mitochondrial respiration (oxygen consumption rate, OCR) measurements in ventricular myocytes from the experimental groups outlined in Fig. 1A using the XF Extracellular Flux Analyzer and the Seahorse Cell Mito Stress assays (illustrated in Fig. 5A).

To date, very few studies measured mitochondrial OCR in adult cardiomyocytes and most used a XFe24 (24 well) extracellular flux analyzer^36,37^. Considering that our experimental setting included many animals in several groups and that it is critical to have good numbers of replicates per animal in the assays, we decided to use a 96 well (XFe96) analyzer for high throughput OCR measurements. First, we optimized the experimental conditions for using the 96 well platform—including myocyte plating density and desired concentrations of FCCP, oligomycin, and rotenone/antimycin A. We tested 5 cell densities (Supplementary Fig. S1A online) and selected 4,800 myocytes/well for subsequent experiments, because OCR values were stable at baseline and showed increased response to FCCP (relative to baseline) from 2400 to 4800/well, whereas FCCP-induced OCR values (relative to baseline) were not further increased and even decreased with higher cell numbers (Supplementary Fig. S1A online). Of 4 FCCP concentrations tested (Supplementary Fig. S1B online), we selected 0.8 µM for subsequent experiments, because it induced the maximal respiration. We found that there was lack of decrease in OCR after oligomycin injection and determined that it was not due to myocyte quality/healthy or insufficient amount of oligomycin (Supplementary Fig. S1C online), which suggests that proton leak is a major oxygen consuming process in isolated adult ventricular myocytes in culture. We also found low basal respiration in these cells that were determined with different and high concentrations of rotenone/antimycin A (Supplementary Fig. S1D online).

Based on these unique features of mitochondrial bioenergetics in isolated adult rat ventricular myocytes as we described above (i.e., low basal respiration, lack of decrease in OCR after oligomycin, and dynamic OCR response to FCCP), it became important and more meaningful to assess OCR on the maximal respiration capacity and spare respiratory capacity (see illustration in Fig. 5A), which are two key parameters reflecting mitochondrial bioenergetic capability of myocytes in respond to increased energetic demands (e.g., during physical exercise). As shown in Fig. 5B-D, both maximal respiration capacity and spare respiratory capacity were significantly reduced in ventricular myocytes from AAC + vehicle group than that from sham + vehicle group, and valsartan had no beneficial effect. In contrast, sacubitril/valsartan improved the AAC-induced reduction in maximal respiration capacity and spare respiration capacity to a level that was not significantly different from sham-operated controls, suggesting that sacubitril/valsartan protects ventricular myocytes from mitochondrial dysfunction in response to persistent LV pressure overload.

### Sacubitril/valsartan is superior to valsartan in reducing mitochondrial superoxide in ventricular myocytes from pressure-overloaded hearts and in Ang II-treated ventricular myocytes in culture

**Figure 6:**
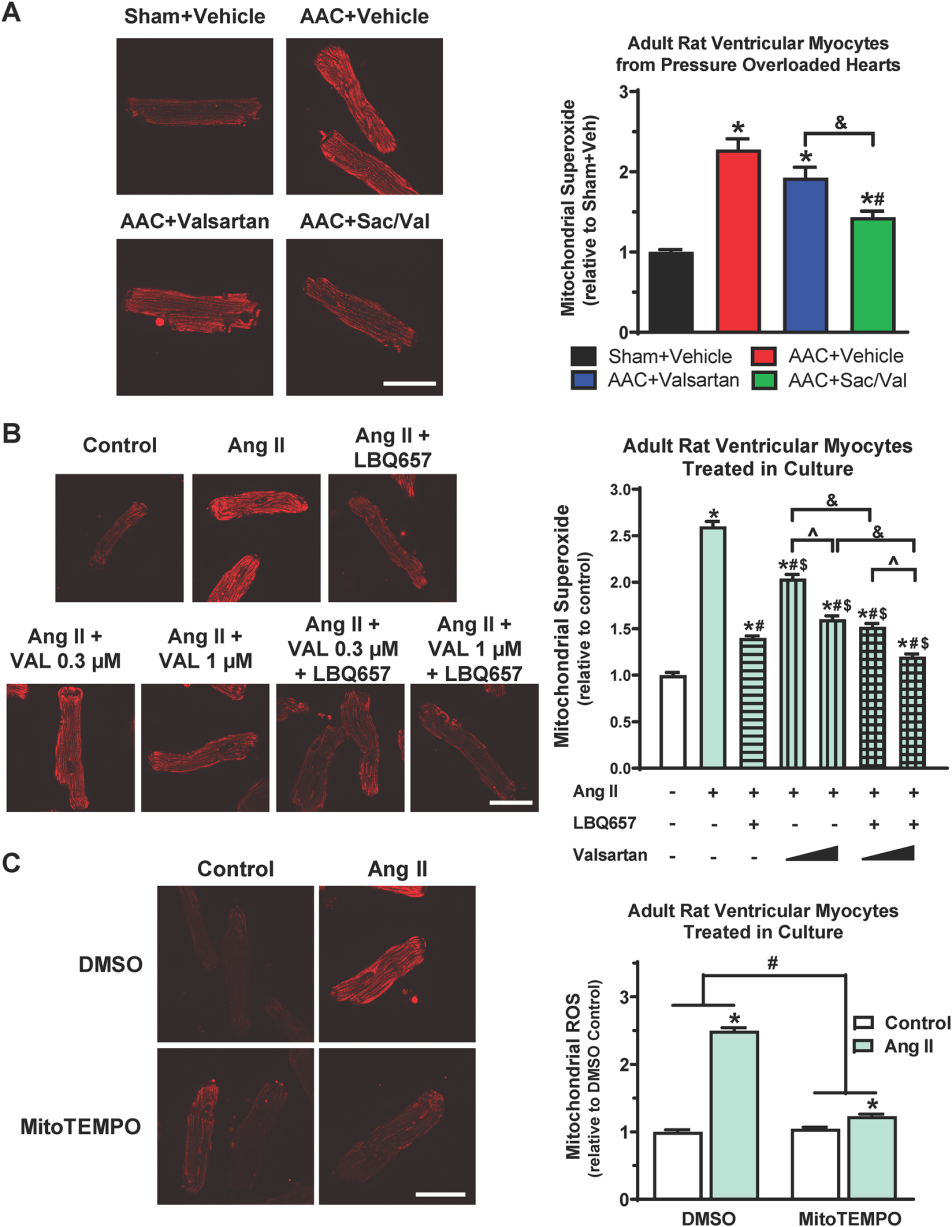
Sacubitril/valsartan is superior to valsartan in reducing mitochondrial superoxide in ventricular myocytes from pressure-overloaded hearts and in Ang II-treated ventricular myocytes in culture. (**A**) Cardiomyocytes isolated from pressure-overloaded hearts after 10-week treatment were stained with MitoSOX Red (5 µM, 15 min) and imaged by confocal microscope. Representative confocal images (60× magnification, scale bar: 50 µm, *left*) and quantitation (*right*) of fluorescent intensities of 100–200 cells averaged for each rat as one biological sample. Mean ± SEM for N = 6–7 rats per group. Sac/Val: Sacubitril/Valsartan. *P < 0.05 versus Sham + Vehicle; ^#^*P* < 0.05 versus AAC + Vehicle; ^&^*P* < 0.05 for the indicated comparison. (**B**) Cardiomyocytes isolated from healthy, untreated rats and pretreated in vitro with LBQ657 (10 µM), valsartan (0.3 or 1 µM) alone or the combination of LBQ657 (10 µM) and valsartan (0.3 or 1 µM) for 1 h before Ang II stimulation (1 µM, 24 h) were stained with MitoSOX Red (5 µM, 15 min) and imaged by confocal microscope. Representative confocal images (60× magnification, scale bar: 50 µm, *left*) and quantitation (*right*) of fluorescent intensities of myocytes treated in vitro. Mean ± SEM for n = 140–200 myocytes per group. *P < 0.05 versus Control. ^#^*P* < 0.05 versus Ang II; ^$^*P* < 0.05 versus Ang II + LBQ657; ^*P* < 0.05 Valsartan at 1 µM versus 0.3 µM; ^&^*P* < 0.05 for the indicated comparison. **(C)** Cardiomyocytes from healthy, untreated rats were pretreated with MitoTEMPO (10 µM, 1 h) before Ang II stimulation (1 µM, 24 h), followed by MitoSOX Red (5 µM, 15 min) staining and confocal imaging. Representative confocal images (60× magnification, scale bar: 50 µm, *left*) and quantitation (*right*) of fluorescent intensities of myocytes. Mean ± SEM for n = 140–200 myocytes per group. *P < 0.05 versus respective control. ^#^*P* < 0.05 for the indicated comparison.

We next asked whether sacubitril/valsartan attenuates mitochondrial oxidative stress in ventricular myocytes from pressure-overloaded hearts. Mitochondrial superoxide levels in myocytes were measured by MitoSOX™ Red labelling and live cell confocal imaging. As shown in Fig. 6A, AAC induced a 2.3-fold increase in mitochondrial superoxide levels that was reduced to 1.9-fold by valsartan (*P* = 0.09) and to 1.4-fold (*P* < 0.001) by sacubitril/valsartan, indicating that sacubitril/valsartan has a superior anti-oxidative effect in pressure overloaded ventricular myocytes than valsartan alone.

We then determined the anti-oxidative effects of sacubitril/valsartan in primary adult ventricular myocytes that were isolated from healthy, untreated rats and stimulated with Ang II in culture. As shown in Fig. 6B, Ang II led a 2.6-fold increase of mitochondrial superoxide level in adult ventricular myocytes in culture, which was reduced to 1.4-fold by LBQ657. Valsartan dose-dependently attenuated Ang II-induced mitochondrial superoxide but was less effective than LBQ657. The combination of valsartan and LBQ657 further significantly augmented the inhibitory effect of valsartan alone, suggesting that sacubitril/valsartan is superior to valsartan alone in reducing Ang II-induced mitochondrial oxidative stress in adult ventricular myocytes.

We also treated adult ventricular myocytes with MitoTEMPO, a specific scavenger of mitochondrial superoxide. MitoTEMPO almost abolished Ang II-induced increase in total cellular superoxide (Fig. 6C), demonstrating that the Ang II-induced increase of superoxide in ventricular myocytes primarily originates from mitochondria and the MitoSOX™ Red specifically labels mitochondrial superoxide. These data also supported our findings that the superoxide specifically labelled by MitoSOX™ Red in ventricular myocytes from pressure-overloaded hearts indeed originated from mitochondria (Fig. 6A).

## Discussion

In this study, we determined the effects of sacubitril/valsartan versus valsartan alone on cardiac remodeling together with mitochondrial function and oxidative stress in pressure overloaded hearts. Our main findings are: (1) Sacubitril/valsartan is superior to valsartan in reducing ventricular fibrosis in response to persistent LV pressure overload; (2) Sacubitril/valsartan is superior to valsartan in reducing Ang II-induced adult ventricular fibroblast activation in culture; (3) Sacubitril/valsartan protects ventricular myocytes from mitochondrial dysfunction in response to persistent LV pressure-overload; (4) Sacubitril/valsartan is superior to valsartan in reducing mitochondrial superoxide in ventricular myocytes from pressure-overloaded hearts and in Ang II-treated ventricular myocytes in culture. To our knowledge, this is the first study suggesting that sacubitril/valsartan in the pressure overloaded hearts is cardioprotective, which is independent of LV unloading via lowering systemic blood pressure.

Sacubitril/valsartan is superior to RAAS inhibition alone in patients with heart failure and preclinical heart failure animal models^15–19^, in which the superiority of sacubitril/valsartan in reducing systemic blood pressure (and thereby unloading of LV) is one of the reasons for the favorable outcomes^38^. In order to study the direct effects of sacubitril/valsartan on the pressure overloaded hearts independent of changes in afterload, we selected AAC surgery to induce LV pressure overload in rats, which is a well-characterized, fixed LV afterload model and has been widely used in studies including testing efficacy of novel therapeutic strategies^22–24^. Given the critical importance of comparable LV afterload among AAC treatment groups in the study, particularly in the comparison of the efficacy between treatments with sacubitril/valsartan and valsartan alone, we used a standard size of vascular clip rather than a ligature to constrict the ascending aorta, which not only increased the consistency of the banding severity but also allowed us to measure the internal area of the clips at the end of the study. Consistent with this notion, Fig. 1B/C demonstrated the high consistency of the internal area of the clips together with the comparable increase in ascending aortic peak blood velocity among the 3 AAC groups, suggesting that the rats in the AAC groups were subjected to a highly comparable increase in LV afterload over the course of the study. We started the treatments 1 week after surgery, because we focused our study on the effects of sacubitril/valsartan as an early intervention in settings of pressure overload. It was reported for this AAC rat model that cardiac hypertrophy develops within 2 weeks after AAC surgery and continues to progress thereafter and that cardiac interstitial fibrosis is observed by 8 weeks after surgery^24^. We thus continued the treatments for a total of 10 weeks (11 weeks after AAC surgery), which was anticipated to be a time point that cardiac remodeling occurred with no signs of ventricular dysfunction yet.

As expected, compared to sham-operated rats, vehicle-treated AAC rats developed characteristic cardiac hypertrophy at the end of the study (11 weeks after surgery), which was evidenced by increased ventricular weight to tibia length ratios and by echocardiographic measurements (Table 1). Through the course of the study, there were no signs of ventricular dysfunction in vehicle-treated AAC rats and the EF in vehicle-treated rats was higher than that in sham-operated rats, suggesting the stage of compensatory cardiac hypertrophy. In comparison to vehicle-treated AAC controls, 10-week treatment with either sacubitril/valsartan or valsartan alone did not alter ventricular hypertrophy development (Fig. 2A) or function (Table 1), suggesting that ventricular hypertrophy is required to compensate the persistently increased LV afterload to maintain cardiac output. Indeed, these observations are expected in absence of LV unloading and are consistent with the findings from many studies on ACE inhibitors^39^, in which ACE inhibitor-induced afterload reduction is believed as the major reason for reduction/regression of cardiac hypertrophy. Using adult ventricular myocytes that were isolated from healthy rats and treated in culture, our data show that valsartan at 1 µM, but not at 0.3 µM, significantly inhibits Ang II-induced increase in ANF mRNA expression (Fig. 3A). While valsartan inhibited Ang II-induced protein synthesis, the effect was partial and showed no significant difference between the two doses tested (Fig. 3B). The partial inhibition of protein synthesis by valsartan may be due to incomplete block of AT_1_ receptors, the presence of autocrine effects, and/or other potential mechanisms (e.g., contribution from activation of AT_2_ receptors). The different inhibitory patterns of valsartan on ANF mRNA expression and protein synthesis indicate that different downstream signaling pathways could be involved in these two responses. Our data also show that the combination of valsartan and LBQ657 does not further enhance the inhibitory effects of valsartan alone on either Ang II-induced ANF mRNA expression or protein synthesis (Fig. 3). Together, these in vitro data suggest modest effects of valsartan on adult ventricular myocyte hypertrophy, for which sacubitril/valsartan is not superior to valsartan alone. Our findings also support the view that afterload reduction is necessary for the anti-hypertrophy effects of sacubitril/valsartan or valsartan in vivo.

In contrast to the effects on hypertrophy, our in vivo data demonstrate that sacubitril/valsartan is superior to valsartan alone in mitigating cardiac fibrosis in pressure-overloaded hearts (Figs. 2B/C). These in vivo observations are supported by in vitro experiments (Fig. 4) that demonstrated a greater inhibitory effect of sacubitril/valsartan than valsartan alone on fibroblast activation, which is a critical event in the development of cardiac fibrosis. The precise mechanism by which the combination of valsartan and LBQ657 is superior to valsartan alone on fibroblast activation warrants further investigation. Cardiac fibrosis has been mainly viewed as a disease modifier secondary to myocyte injury; however, our findings that sacubitril/valsartan represses cardiac fibrosis without changes in cardiac hypertrophy suggest that cardiac fibrosis could also be an independent event in pressure overload-induced cardiac remodeling before cardiac decompensation. To date, beneficial effects of sacubitril/valsartan on cardiac fibrosis reduction were majorly investigated in right ventricles of pulmonary hypertension models^40^ and LV heart failure models induced by various stimuli such as reperfusion injury^17^, myocardial infarction^29^, and pressure overload^41^. Our findings are not only consistent with these studies, but also suggest that sacubitril/valsartan can inhibit the development of cardiac fibrosis prior to occurrence of cardiac decompensation and is superior to treatment with valsartan alone.

Mitochondria are the main energy powerhouses of cells including cardiac myocytes and can carry out important cellular processes^42,43^. Because of their significance, mitochondrial dysfunction has been linked to several cardiovascular disorders, including hypertension and ischemia cardiomyopathy^44,45^, in which they promote the progression of the pathological conditions. Studies documented that mitochondrial dysfunction and increased oxidative stress are also the primary factors in pathological stimuli-induced cardiac dysfunction and its progression^46,47^. Reduction of LV transmural differences in mitochondrial respiratory chain activity and its associated increase of oxidative stress was also reported in pressure overload-induced cardiac hypertrophy^48^. In this study, our data (Fig. 5) showed that AAC causes significant reductions in mitochondrial maximal respiration capacity and spare respiratory capacity, suggesting low capability of cardiac myocytes in generation of ATP to respond to an increased energetic demanding in pressure-overloaded hearts. Importantly, our data also showed that sacubitril/valsartan is able to improve AAC-induced reductions of mitochondrial maximal respiration capacity and spare respiratory capacity (Fig. 5), suggesting a critical beneficial effect of sacubitril/valsartan on mitochondrial bioenergetics. The importance of the mitochondrial spare capacity in cardiac myocytes was also suggested by the study from Hill et al.^49^, showing that neonatal rat ventricular myocytes have a substantial bioenergetic reserve capacity under basal conditions and that exhaustion of this reserve capacity results in inhibition of respiration concomitant with protein modification and cell death. Their data also suggest that mitochondrial reserve capacity is a sensitive measure of cellular stress and can be used to assess and predict the response of cells to stress. Recently, exercise intolerance was reported in rats with pressure overload-induced heart failure and can be improved by sacubitril/valsartan treatment^50^. With our findings, we speculate that protecting mitochondrial respiration capacities by sacubitril/valsartan may also play an important role in the observed improvement of exercise tolerance. Of note, we observed low basal respiration and lack of decrease in OCR after oligomycin in isolated adult cardiac myocytes in culture, which is consistent with the report from others^36,37^. Additionally, we demonstrate and provide optimization data for the first time to facilitate use of a 96 well format for high throughput mitochondrial respiration (e.g., OCR) measurement in adult cardiac myocytes for future studies.

Oxidative stress is related to an excess production of reactive oxygen species (ROS) and mitochondria are the primary source of ROS in cardiac myocytes^43^. Our study reveals that sacubitril/valsartan is superior to valsartan in attenuating mitochondrial superoxide in cardiac myocytes from pressure-overloaded hearts (Fig. 6A). Using adult ventricular myocytes in culture, we show direct evidence that LBQ657 alone effectively attenuates Ang II-stimulated increase in mitochondrial superoxide and that the combination of LBQ657 and valsartan is superior to valsartan or LBQ657 alone (Fig. 6B). Our findings thereby suggest that sacubitril/valsartan is superior to valsartan in protecting cardiac myocytes from oxidative stress. We also would like to highlight here that, similar as the beneficial effects on cardiac fibrosis, the beneficial effects of sacubitril/valsartan on mitochondrial function and oxidative stress in pressure overloaded hearts appear to occur independent of the development of cardiac hypertrophy and can happen during the early intervention.

Together, this study provides direct evidence for and novel mechanistic insights on the superior cardioprotective effects of early intervention with sacubitril/valsartan in the setting of pressure overload: sacubitril/valsartan is superior to valsartan in reducing cardiac fibrosis and myocyte oxidative stress and improves myocyte mitochondrial function independent of afterload reduction. The combination of valsartan and LBQ657 is also superior to valsartan alone in inhibiting Ang II-induced adult ventricular fibroblast activation in culture. This study warrants further research to elucidate the precise mechanism underlying sacubitril/valsartan-mediated mitochondrial regulation, a critical aspect in cardiomyocyte injury and dysfunction in response to various cardiac stress including LV pressure overload. In this study we utilized only male rats. Considering that there are some conflicting data on whether the response to sacubitril/valsartan varies by sex^15,40,51,52^, further studies may be needed to investigate if sex can act as an important variable in the model system we use. We used young rats to start the procedure of ascending aortic constriction with the goal to progressively increase LV afterload (see model description) and to mimic chronic hemodynamic stress or load seen in cardiac patients. It is increasingly appreciated that aging is associated with increased cardiac fibrosis as well as mitochondrial dysfunction and oxidative stress in myocardium^53,54^. Therefore, we speculate that the cardioprotective effects of early intervention with sacubitril/valsartan on pressure overloaded hearts in older rats could be more pronounced than that in young rats in aspects including improvement of cardiac fibrosis and mitochondrial function, but further research is needed to substantiate that. To assess the clinical relevance of our findings, future studies are also needed to determine the cardioprotective effects of sacubitril/valsartan and its superiority over RAAS inhibition alone in patients with LV pressure overload. It is particularly intriguing to know whether sacubitril/valsartan can be a novel intervention in patients with severe aortic stenosis and preserved LV function (who are not yet candidates for aortic valve replacement) to delay the deleterious effects of fixed afterload and thereby can improve outcomes after surgical or transcatheter aortic valve replacement. It is also quite intriguing to know whether sacubitril/valsartan can be a novel intervention in patients with resistant hypertension to delay the occurrence of cardiac dysfunction and failure.

## Supplementary Information

Supplementary Information.

## Data Availability

The data that support the findings of this study are included in this published article (and its Supplementary Information files).
