# Supplementary material for: Cardioprotective effects of early intervention with sacubitril/valsartan on pressure overloaded rat hearts
Source: Sci Rep. 2021 Aug 16;11:16542. doi: 10.1038/s41598-021-95988-3 (PMC8368201; doi:10.1038/s41598-021-95988-3)
Supplement: Supplementary file 1 — Supplementary Information. [file 41598_2021_95988_MOESM1_ESM.pdf]

Online Supplementary Material

(Supplementary Figure S1 & Table S1)

Cardioprotective Effects of Early Intervention with Sacubitril/Valsartan on Pressure Overloaded Rat Hearts

Xiaofei Li [a, c], Julie Braza [b, c], Ulrike Mende [a, c], Gaurav Choudhary [a, b, c], Peng Zhang [a, b, c*]

[a] Cardiovascular Research Center, Lifespan Cardiovascular Institute, Rhode Island Hospital, Providence, RI

[b] Vascular Research Laboratory, Providence VA Medical Center, Providence, RI

[c] Department of Medicine, Alpert Medical School of Brown University, Providence, RI

***Corresponding author**

Supplementary Figure S1 and Legends

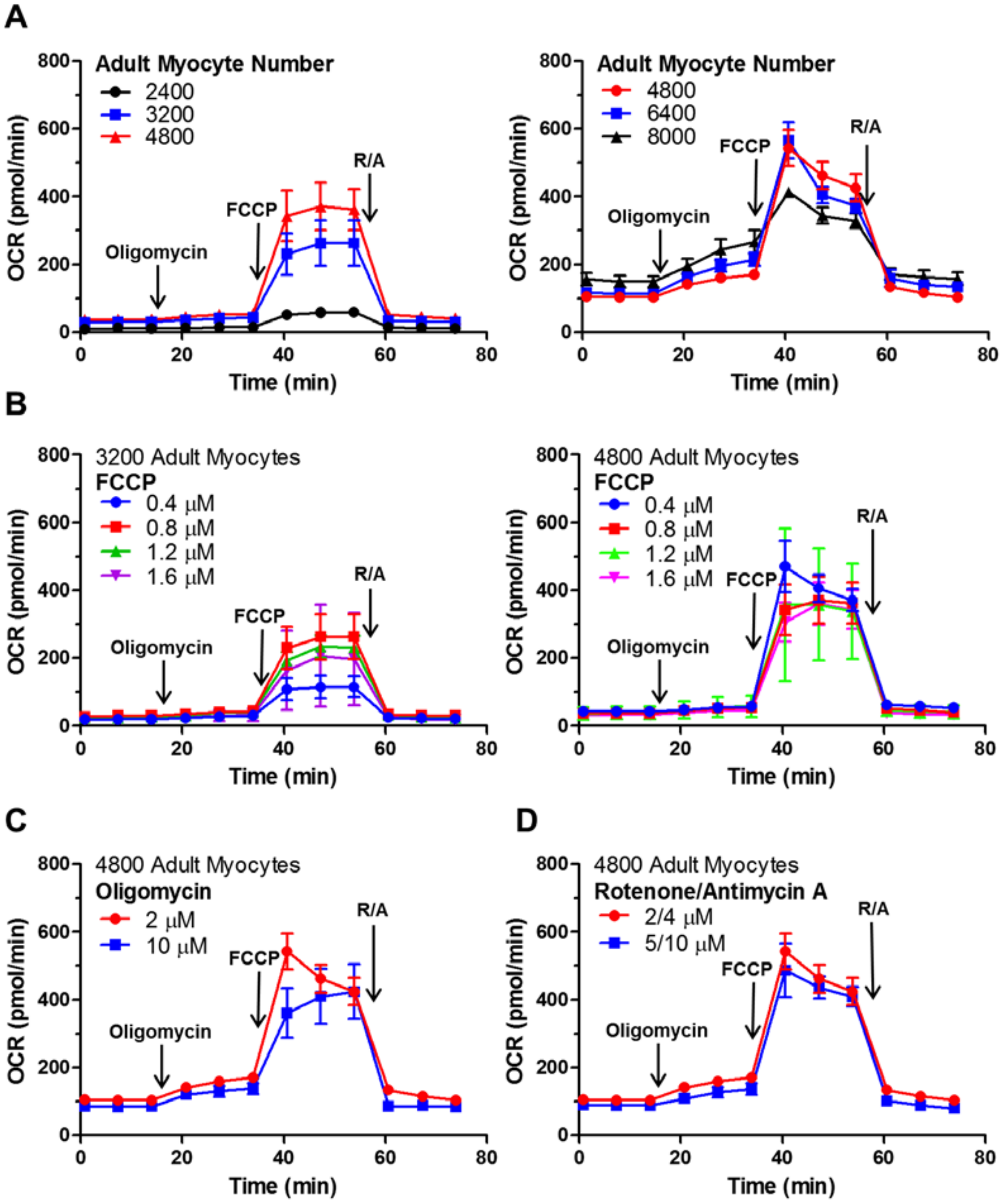

Figure S1: Optimization of adult cardiomyocyte number, FCCP, Oligomycin, and Rotenone/Antimycin A (R/A) concentration for Seahorse mitochondrial respiration measurements. Ventricular myocytes isolated from healthy, untreated adult rats were plated into Seahorse XFe96 V3 PS cell culture microplates. Mitochondrial respiration was measured after 24 hrs. **(A)** Effect of myocyte numbers on OCR (*left*: 2400, 3200, and 4800/well; *right*: 4800, 6400, and 8000/well. Oligomycin: 2

μM; FCCP: 0.8 μM; Rotenone/Antimycin A: 2/4 μM. **(B)** Effect of FCCP concentration (0.4, 0.8, 1.2, and 1.6 μM) on OCR at indicated myocyte numbers (*left*: 3200/well; *right*: 4800/well). Oligomycin: 2 μM; Rotenone/Antimycin A: 2/4 μM. **(C)** Effect of oligomycin concentration (2 and 10 μM) on OCR at 4800 myocytes/well. FCCP: 0.8 μM; Rotenone/Antimycin A: 2/4 μM. **(D)** Effect of Rotenone/Antimycin A concentration (2/4 and 5/10 μM) on OCR at 4800 myocytes/well. FCCP: 0.8 μM; Oligomycin: 2 μM. Mean±SEM. n=5-6 determinations per group.

Supplementary Table S1

Table S1. Body and heart weights of operated rats after 10-week treatment with vehicle (water), valsartan (31 mg/kg/day), and sacubitril/valsartan (68 mg/kg/day).

Parameter	Sham+Vehicle (N=8)	AAC+Vehicle (N=10)	AAC+Valsartan (N=11)	AAC+Sac/Val (N=11)
**BW (g)**	461±11	439±8	428±12	422±6*
**HW (mg)**	1144±21	1636±105*	1587±61*	1609±70*
**VW (mg)**	1066±21	1507±77*	1466±55*	1482±62*
**TL (mm)**	43.8±0.4	43.9±0.4	43.9±0.3	43.7±0.3
**VW/BW (mg/g)**	2.3±0.1	3.4±0.1*	3.4±0.1*	3.5±0.1*
**VW/TL (mg/mm)**	24.4±0.4	34.3±1.6*	33.4±1.2*	33.9±1.4*

Sac/Val: Sacubitril/Valsartan; BW: body weight; HW: heart weight; VW: ventricular weight; TL: tibia length. Mean±SEM. * $P<0.05$ vs. Sham+Vehicle.
